# Recovery of Non-Ferrous Metal from Metallurgical Residues

**DOI:** 10.3390/ma16216943

**Published:** 2023-10-29

**Authors:** Guo Chen

**Affiliations:** Kunming Key Laboratory of Energy Materials Chemistry, Yunnan Minzu University, Kunming 650500, China; guochen@kust.edu.cn; Tel.: +86-871-6591-0017

Non-ferrous metals and alloys are essential resources for the development of modern industries. With the depletion of natural minerals, the recovery of non-ferrous metals from metallurgical residues attracts researchers from multidisciplinary areas. Ideas for new recovery routes reduce the pressure on natural resources and the environment, thus enabling better manufacturing sustainability. This Special Issue primarily considers papers focused on the theoretical and engineering aspects of processing metal recovery from metallurgical residues. The purpose of the current editorial is to briefly summarize the publications included in this Special Issue.

Dzinomwa et al. examined the historic slag produced from a smelter in Namibia, which accumulated over decades of its operating life [1]. Based on the results, approximate conditions under which the different slag phases were formed were estimated, and the recovery routes for the various metals were proposed. Zheng et al. summarize the physicochemical characteristics and general processing methods of coal gangue and fly ash and review the progress in the application of porous materials prepared from these two solid wastes in the fields of energy and environmental protection, including the following: the adsorption treatment of heavy metal ions, ionic dyes, and organic pollutants in wastewater [2]. Tian et al. studied the application of microwave technology in recovering valuable metals from the leaching residue produced from zinc production [3]. Zheng et al. studied the process conditions of recycling Zn from metallurgical slag and dust material leaching using ammonium acetate (NH_3_-CH_3_COONH_4_-H_2_O) [4]. The influences of the liquid/solid ratio, stirring speed, leaching time, and total ammonia concentration as well as the interactions between these variables on the Zn effective extraction rate during the ammonium acetate leaching process were investigated. They also proposed an experimental study on ultrasound-enhanced sulfuric acid leaching for zinc extraction from zinc oxide dust [5]. Ultarakova et al. present studies on the ammonium fluoride processing of dust from the reduction smelting of ilmenite concentrate with silicon separation to obtain titanium dioxide [6]. Optimal conditions for pyrohydrolysis of titanium fluorides were determined. The effects of temperature and duration on the process were studied.

Regarding the preparation of advanced materials, the following articles are included in this Special Issue. Wang et al. prepared micron-sized silver particles using the chemical reduction method by employing a Y-type microjet reactor, silver nitrate as the precursor, ascorbic acid as the reducing agent, and gelatin as the dispersion at room temperature [7]. Using a microjet reactor, the two reaction solutions collide and combine outside the reactor, thereby avoiding microchannel obstruction issues and facilitating a quicker and more convenient synthesis process. The resistivity of conductive silver paste prepared with the as-synthesized spherical silver particles was also discussed in detail [8]. Orda et al. contribute to the technique development of purification commercial rhenium salts [9]. The adsorption behavior of Sc on the surface of kaolinite (001) was investigated using the density functional theory via the generalized gradient approximation plane-wave pseudopotential method by Zhao et al. [10]. Zhang et al. researched the thermal deformation behavior of titanium ingots prepared using EB furnaces, which can reduce the cost of titanium production [11].

Some of the attempts at optimizing the traditional metallurgical process are also presented in this Special Issue, the research method of which can be referenced for the slag treatment. Research on limonite pellet technology is crucial for iron making as high-grade iron ore resources decline. However, pellets undergo rigorous mechanical actions during production and use. Yan et al. prepared a series of limonite pellet samples with varying ratios and measured their compressive strength. Artificial neural networks (ANN) predicted the compressive strength of humic acid and bentonite-based pellets, establishing the relationship between input variables (binder content, pellet diameter, and weight) and the output response (compressive strength). Integrating pellet technology and machine learning drives limonite pellet advancement, contributing to emission reduction and environmental preservation [12]. Zhang et al. used a pelletizing method to enhance the subsequent iron-making process by applying Guisha limonite, with advantages including large reserves and low price [13]. The purpose is to provide an alternative for the sinter, thus reducing the greenhouse gas emissions during the iron-making process. A multivariate regression model for estimating the compressive strength of pellets was developed using the Box–Behnken experimental methodology, where the relevant factors were the roasting temperature, pellet diameter, and bentonite content.

For steel making, electromagnetic stirring (M-EMS) has been extensively applied in continuous casting production to reduce the quality defects of casting billets. To investigate the effect of continuously casting electromagnetic stirring on billet segregation, a 3D multi-physics coupling model was established to simulate the internal heat, momentum, and solute transfer behavior in order to identify the effect of M-EMS on the carbon segregation of a continuous casting square billet [14]. The quality of the bloom will be impacted by the non-metallic impurities in the molten steel in the tundish, which will reduce the plasticity and fatigue life of the steel. Yi et al. established a six-flow double-channel T-shaped induction heating tundish mathematical model. The effects of induction heating conditions on the removal of inclusions in the tundish were investigated, and the impact of various inclusion particle sizes on the removal effect of inclusions under induction heating was explored [15]. The effect of Cu on the formation of reversed austenite in super martensitic stainless steel was investigated by Jiang et al. using X-ray diffraction (XRD), a transmission electron microscope (TEM), and an energy-dispersive spectrometer (EDS) [16].
